# Optically Stimulated Luminescence Sensitivity of Quartz for Provenance Analysis

**DOI:** 10.3390/mps3010006

**Published:** 2020-01-13

**Authors:** André Oliveira Sawakuchi, Fernanda Costa Gonçalves Rodrigues, Thays Desiree Mineli, Vinícius Ribau Mendes, Dayane Batista Melo, Cristiano Mazur Chiessi, Paulo César Fonseca Giannini

**Affiliations:** 1 Luminescence and Gamma Spectrometry Laboratory (LEGaL), Instituto de Geociências, Universidade de São Paulo, São Paulo 05508-080, Brazil; cgr.fernanda@gmail.com (F.C.G.R.); thaysdesiree@gmail.com (T.D.M.); pcgianni@usp.br (P.C.F.G.); 2 Institute of Marine Sciences, Federal University of São Paulo, Santos 11015-020, Brazil; vrm.unifesp@gmail.com (V.R.M.); d.batista.melo@hotmail.com (D.B.M.); 3 School of Arts, Sciences and Humanities, University of São Paulo, São Paulo 03828-000, Brazil; chiessi@usp.br

**Keywords:** sediment provenance, quartz fingerprint, luminescence, source-to-sink system

## Abstract

Finding the source or provenance of quartz grains occurring in a specific location allows us to constrain their transport pathway, which is crucial information to solve diverse problems in geosciences and related fields. The optically stimulated luminescence (OSL) sensitivity (light intensity per unit mass per unit radiation dose) has a high capacity for discrimination of quartz sediment grains and represents a promising technique for provenance analysis. In this study, we tested the use of quartz OSL sensitivity (ultraviolet emission) measured under different preheating temperatures and with blue light stimulation at room temperature (~20 °C) for sediment provenance analysis. Quartz OSL sensitivity measured at 20 °C is positively correlated with the sensitivity of an OSL signal measured using procedures (preheat at 190 °C for 10 s, blue stimulation at 125 °C and initial 1 s of light emission) to increase the contribution of the fast OSL component, which has been successfully applied for sediment provenance analysis. The higher OSL signal intensity measured without preheating and with light stimulation at room temperature allows the use of lower given doses, thus reducing measurement time. Additionally, the OSL sensitivity measured at 20 °C in polymineral silt samples of a marine sediment core is also suitable for provenance analysis, as demonstrated by comparison with other independent proxies. OSL signals obtained through light stimulation at room temperature have thus the potential to considerably expand measurement possibilities, including in situ measurements using portable OSL readers.

## 1. Introduction

Mineral grains resting on or near Earth’s surface can be dispersed through multiple ways, such as by landslides induced by earthquakes [1] or extreme rainfall events [2], winds [3], rivers [4], coastal waves, or tidal currents [5], soil-living insects [6], and diverse human activities [7]. Tracking the primary or previous source of mineral grains allows for the reconstruction of varied events, ranging from past climate changes [8] to a pathway taken by a crime suspect [9]. Thus, methods to find the source or the provenance of mineral grains have several applications in geosciences and related fields. Quartz (SiO_2_) is the most frequent mineral in sediments [10], and it is widespread over the Earth’s surface, especially in the sand (63–2000 µm) and silt (4–63 µm) fractions. Thus, quartz is a valuable material in provenance studies due to its ubiquitous occurrence and high stability in most Earth surface settings. However, quartz grains are poorly discriminated by methods commonly used for provenance analysis of sediments, which are based on the chemical [5] or mineralogical composition of sediment mixtures or on chemical variations of specific minerals [11]. The optically stimulated luminescence (OSL) is the light arising from charges trapped in crystal lattice defects when quartz is exposed to ionizing radiation and subsequently released for recombination when quartz is exposed to a stimulation light [12]. The OSL sensitivity is defined as the intensity of light emitted per unit mass of the material and unit radiation dose (photon counts Gy^−1^ mg^−1^). The quartz OSL sensitivity ranges over five orders of magnitude [13,14] and can be measured in different types of sediment or sedimentary rock samples. Luminescence readers allow performing automated OSL measurements on aliquots (amount of grains) of silt, very fine to fine sand (62–250 µm), single grains of fine sand, or slices of sedimentary rocks [15,16,17] in a fast and low-cost way. Quartz has relatively low OSL sensitivity when crystallized in its parent igneous, hydrothermal, or metamorphic rocks [13,18], but it can be subsequently sensitized by artificial or natural heating [19] or illumination-irradiation cycles [20]. In nature, the OSL sensitization occurs after quartz is released from its parent rocks and is transported as a grain in sedimentary systems [13]. The OSL sensitization during the transport of quartz as sediment grain is attributed to repeated cycles of bleaching and irradiation [20,21,22]. Despite the fact that the increase of quartz OSL sensitivity due to bleaching–irradiation cycles is well demonstrated by laboratory experiments, e.g., [19,22], it is not conclusive if the sensitization is related with changes in the concentration of charge traps, recombination processes, or both [14,20]. However, this condition does not hinder the use of quartz OSL sensitivity for sediment provenance analysis, e.g., [21]. Previous studies to apply quartz OSL sensitivity in provenance analysis, e.g., [22,23,24] focused on the initial 1 s of the OSL decay curve obtained by constant energy blue light stimulation at 125 °C after heating the sample at temperatures from 160 to 260 °C, to eliminate unstable signals [25]. The OSL signal measured under this condition is presumably dominated by the fast OSL component [26], but quartz with low OSL sensitivity occurring in sediments around young mountain ranges can show the initial 1 s of light emission with significant contribution of medium and slow components [14]. Despite the successful use of the unstable 110 °C thermoluminescence (TL) peak of quartz in provenance analysis of coastal and marine sediments [27,28], little is known about the suitability of using unstable OSL signals for sediment-fingerprinting purposes. In this study, we performed experiments to improve measurements of quartz OSL sensitivity for provenance analysis. Experiments aimed to simplify protocols for OSL sensitivity measurements, including evaluation of heavy minerals contamination and whether thermal treatments to eliminate unstable OSL signals are necessary. Our experiments show that quartz OSL sensitivity measured without preheating and with optical stimulation at room temperature (~20 °C) can be used for provenance analysis in the same way as the OSL measured with preheating and light stimulation at 125 °C [21,22,23,28]. This expands the measurements conditions of quartz OSL sensitivity to in situ measurements in sediment cores and outcrop sedimentary successions, as well as in thermally sensitive materials hosting quartz.

## 2. Materials and Methods

OSL sensitivity measurements were carried out on quartz crystals from hydrothermal veins and metamorphic rocks (blue schist) and on quartz sand grains extracted from sediments from different geological settings (Table 1). Sediment samples were purposely selected to cover a wide range of quartz OSL sensitivity, which was appraised in a previous study [14]. Additionally, 16 samples from marine core GeoB16206-1 [29] were analyzed to further the application of OSL sensitivity provenance analysis to fine-grained (silt) sediments.

Rock samples (IP22B and BS223) were crushed using a hammer, and quartz-crystal fragments (1–5 mm) were handpicked for milling, manually, using a pestle and mortar. Afterward, these samples were submitted to sieving and chemical treatments, in the same way as sand samples, to isolate quartz grains. The procedures to obtain pure quartz concentrates for luminescence measurements consisted of: (1) wet sieving for isolation of 180–250 or 63–180 µm grain fraction; (2) treatment with H_2_O_2_ 35% (not applied to quartz from rocks) and HCl 10%, to eliminate organic matter and carbonate minerals; (3) density separation with lithium metatungstate (LMT) solutions at densities of 2.75 and 2.62 g/cm^3^, to isolate quartz grains from heavy minerals and feldspar grains; (4) treatment with HF 40% for 40 min, to etch the outer rind of the quartz grains and to remove remaining feldspar; and (5) washing with distilled water and another sieving for isolation of the 180–250 or 63–180 µm grain fractions. The purity of quartz concentrates was tested by using infrared stimulation. Additional HF 5% (24 h) treatments were applied if a significant infrared stimulated luminescence signal was observed. It is also recommended to apply an additional HCl 10% wash, to eliminate possible fluorides formed as product of HF treatments. This step is especially important for samples rich in feldspar. For the fine-grained sediments of core GeoB16206-1, sample preparation [28] included the following: (1) sample drying at 60 °C; (2) weighing of 0.5 g of each sample; (3) treatment with H_2_O_2_ 27% and HCl 10%, to remove organic matter and carbonate minerals; and (4) addition of acetone to the content until 5 mL.

Two samples of Holocene coastal sands (L0387 and L0393) from Southern Brazil (Santa Catarina) were used to obtain heavy mineral concentrates, using the following procedures: (1) dry sieving to isolate the 63–125 µm grain-size fraction; (2) heavy liquid separation with bromoform, at a density of 2.85 g/cm^3^, to isolate heavy and light minerals; (3) separation of magnetic and nonmagnetic heavy minerals, using a hand-magnet; and (4) weighing of heavy minerals. Aliquots of magnetic and nonmagnetic heavy minerals were used for luminescence measurements. Transparent nonmagnetic heavy mineral types were also identified and quantified under polarized light optical microscopy. The 63–125 µm fraction was chosen for heavy mineral analysis, to facilitate mineral identification under the optical microscope. However, the heavy mineral types from this grain-size fraction are representative of heavy minerals occurring in the grain-size fraction used in this study for the luminescence analysis of quartz sand grains (63–180 and 180–250 µm).

Luminescence measurements were carried out in the Luminescence and Gamma Spectrometry Laboratory at the Institute of Geosciences of the University of São Paulo. Measurements of quartz concentrates in the sand fraction were performed in a Risø TL/OSL DA-20 reader, equipped with a built-in beta source (^90^Sr/^90^Y; dose rate of 0.0981 Gy s^−1^ for 11.65 mm diameter stainless-steel cups), blue (470 nm, maximum power of 80 mW/cm^2^) and infrared (870 nm, maximum power of 145 mW/cm^2^) LEDs for stimulation, and Hoya U-340 filter for light detection in the ultraviolet band. Fine-grained polymineral sediments from core GeoB16206-1 were measured in a Lexsyg Smart TL/OSL reader, equipped with blue (458 nm, maximum power of 100 mW/cm^2^) and infrared (850 nm, maximum power of 300 mW/cm^2^) LEDs, filters for light detection in the ultraviolet band (365 nm, U340 + BP 365/50), and beta radiation source (^90^Sr/^90^Y), with a dose rate of 0.116 Gy s^−1^ for stainless steel discs.

In all experiments, the OSL sensitivity (blue stimulation, BOSL) was calculated by using the integral of the first 1 s of light emission, minus the last 10 s as background. In experiments carried out with quartz in the sand grain size, the OSL sensitivity calculation applied normalization by dose and aliquot mass (photon cts Gy^−1^ mg^−1^). In the first experiment, OSL sensitivity was also calculated as a percentage of the initial 1 s of light emission in relation to the total OSL emission (%BOSL). This approach has been used to minimize the effect of aliquot mass variation on the OSL sensitivity [23]. However, this approach limits the range of sensitivity variation (0–100%) and reduces the capacity of sediment discrimination. The OSL sensitivity of fine-grained aliquots from core GeoB16206-1 was only calculated as a percentage of the first second of light emission in relation to the total emission (%BOSL). OSL decay curves for sensitivity calculation were analyzed by using the software Luminescence Analyst v4.31.9 [31].

For each experiment, three aliquots per sample were prepared by mounting quartz or heavy mineral grains in stainless-steel cups for measurements in the Risø TL/OSL DA-20 reader. An acrylic plate with a circular microhole of 1470 μm diameter, with 1860 μm depth, was used to prepare aliquots with similar volume [23], corresponding to 150–200 grains (180–250 μm grain size) and ensuring a monolayer of grains onto cups or discs. Each aliquot was weighed for mass normalization of luminescence signals. Aliquots of fine-grained sediments of the marine sediment core were mounted by using four drops of acetone–sediment solution over stainless-steel discs, for measurements in the Lexsyg Smart TL/OSL reader. Mounting of silt aliquots followed standard procedures used for luminescence dating [32].

The first experiment comprised the measurement of OSL sensitivity of quartz and heavy mineral concentrates, using the same protocol described in previous work [23] (Table 2). The first OSL stimulation (step 1) bleaches natural signals. A relatively high given dose of 50 Gy was used to guarantee measurable signals in low-sensitivity quartz and heavy mineral aliquots. After the given dose (step 2), the infrared stimulation (step 4) aims to identify quartz aliquots contaminated by feldspar or to bleach feldspar grains and measure quartz in polymineral aliquots [24]. The final blue stimulation (step 5) is used to determine the quartz OSL sensitivity (pure quartz aliquots) or a quartz-dominated OSL sensitivity (polymineral aliquots). Pure quartz aliquots with significant infrared-stimulated luminescence signal were not used for sensitivity calculation. The OSL sensitivity measured using step 5 of the protocol in Table 2, subsequent to a preheating at 190 °C for 10 s (in step 3) and using blue stimulation at 125 °C, is termed “BOSL_F_”; this signal presumably has a higher contribution of the fast OSL component [23].

The second experiment evaluated the influence of the preheat temperature on the OSL sensitivity. In this experiment, the same protocol described in Table 2 was applied, but using preheat temperatures of 50, 100, 150, 200, 300, and 400 °C, to measure the OSL sensitivity in samples IP22B, L0017 and L0229, which have low, medium, and high BOSL_F_ sensitivities, respectively (Table 1). The OSL sensitivity from this experiment is also named BOSL_F_. However, it is important to note that, despite the fact that higher preheating temperatures increase the fast-to-slow components ratio, the dominance of a fast OSL component in the BOSL_F_ signal is sample-dependent [26]. The OSL sensitivity from other measurement conditions applied in this study is specified accordingly.

The third experiment was designed to evaluate the measurement of quartz OSL sensitivity, without preheating and with stimulation at room temperature. Six quartz samples with low (IP22B and BS223), medium (L0017 and L0698), and high (L0001 and L0229) BOSL_F_ sensitivity were used for this test. The sequence of procedures (Table 3) comprises a longer bleaching with blue-light illumination for 500 s (step 1), a given beta dose of 50 Gy (step 2), no pause or pause of 16 h to eliminate unstable signals (step 3), OSL stimulation using blue light at 20 °C (step 4), and a second OSL stimulation using blue light at 125 °C (step 5). The longer bleaching (500 s) was applied because illumination was performed at room temperature, while the protocol from Table 2 used a bleaching at 125 °C. The infrared stimulation step was not included, in order to reduce protocol time for measurement of pure quartz aliquots. The pause before blue stimulation aimed to evaluate any effect of the 110 °C TL trap. The effect of a 16 h pause is similar to heating the sample at 125 °C for 10 s [20]. Thus, the pause was applied to eliminate the influence of the 110 °C TL trap on the further OSL measurement at 20 °C (BOSL_20°C_) (step 4). Measurements conduced with a 16 h pause were used to validate measurements carried out with no pause. The blue stimulation at 125 °C (step 5) was performed to evaluate if the OSL sensitivity (BOSL_125°C_) can be measured twice without signal reset and dosing.

The sediment samples from marine core GeoB16206-1 were measured following the protocol described in Table 4. The BOSL_20°C_ sensitivity for these samples were calculated as the percentage of initial 1 s of light emission in relation to the total OSL emission (0–100 s). Then, the BOSL_20°C_ was normalized by subtracting the average sensitivity and dividing by the standard deviation of all samples. This normalization was applied in order to compare the BOSL_20°C_ with BOSL_F_ values [28].

## 3. Results

**Figure 1:**
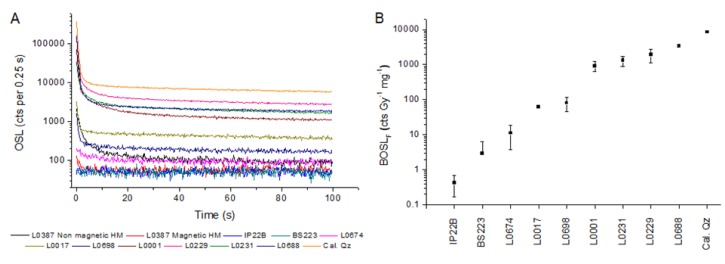
(**A**) OSL decay curves (step 5 of the protocol in Table 2) of quartz with different sensitivities. OSL decay curves of nonmagnetic and magnetic heavy minerals are also shown for comparison. Aliquots irradiated with 50 Gy. (**B**) Range of BOSL_F_ sensitivity comprised in the studied quartz samples. Each sample is represented by the average of three aliquots. Error bars correspond to the standard deviation. Sample type is described in Table 1.

BOSL_F_ sensitivities of the studied quartz samples range in five orders of magnitude, from 0.42 ± 0.25 to 8565.00 ± 497.76 cts Gy^−1^ mg^−1^ (Figure 1 and Table 5). The lowest BOSL_F_ sensitivity occurs in quartz from a hydrothermal vein (IP22B), and the highest sensitivity is observed in artificially sensitized quartz (CalQz). Magnetic heavy minerals, mainly represented by magnetite, have OSL sensitivity between 0.63 ± 0.29 and 1.19 ± 0.65 cts Gy^−1^ mg^−1^. The OSL sensitivity of nonmagnetic heavy minerals varied between 20.80 ± 6.00 and 94.77 ± 103.03 cts Gy^−1^ mg^−1^. Heavy minerals correspond to 18% and 12% (weight percent) of the 63–125 µm grain-size fraction in samples L0387 and L0393, respectively. The nonmagnetic and transparent heavy minerals in the studied samples include zircon (75–76%), rutile (6–7%), tourmaline (5–6%), kyanite (4%), staurolite (4%), epidote (1–3%), monazite (<1%), sillimanite (<1%), hornblende (<1%), and garnet (<1%). Figure 1 shows OSL decay curves of quartz, magnetic heavy minerals, and nonmagnetic heavy minerals for comparison.

**Figure 2:**
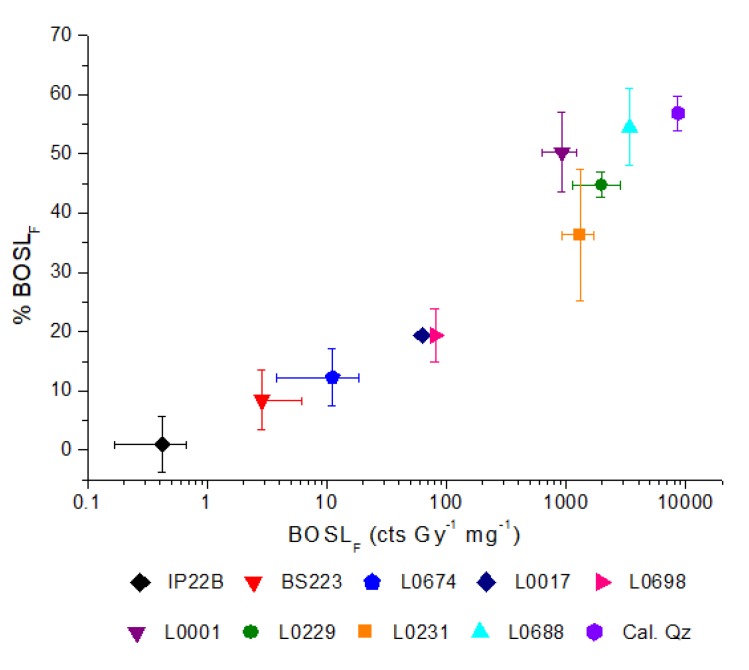
Quartz OSL sensitivity represented by the percentage of the initial 1 s of light emission (%BOSL_F_) compared to the absolute quartz BOSL_F_ sensitivity (normalized by dose and aliquot mass). Each sample is represented by the average of three aliquots. Error bars correspond to the standard deviation.

The BOSL_F_ sensitivity normalized by mass and dose is correlated with the BOSL_F_, represented as a percentage of the total OSL emission (%BOSL_F_). The advantage of BOSL_F_ over %BOSL_F_ is its larger amplitude of variation and, then, the higher capacity of quartz discrimination, especially among higher OSL sensitivity quartz (Figure 2).

**Figure 3:**
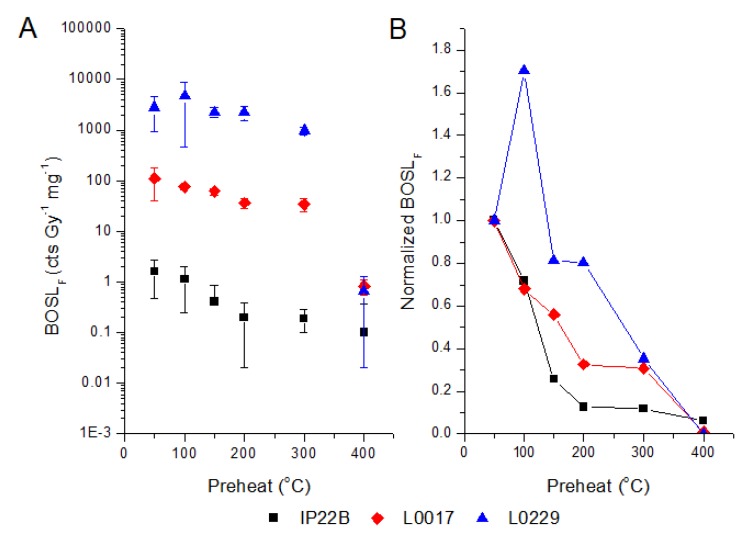
(**A**) Variation of BOSL_F_ sensitivity (step 5 of Table 2), measured using preheat temperatures from 50 to 400 °C. (**B**) Variation of BOSL_F_ sensitivity (preheat from 50 to 400 °C) normalized by the BOSL_F_ sensitivity measured with preheat at 50 °C. Sensitivity values correspond to the average of three aliquots. Error bars are the standard deviation. Sample type is described in Table 1. BOSL_F_ sensitivity values are shown in Table 6.

The BOSL_F_ sensitivity decreases with the increase of preheat temperature, but sensitivity differences among samples are not affected by the preheat temperature (Figure 3 and Table 6).

**Figure 4:**
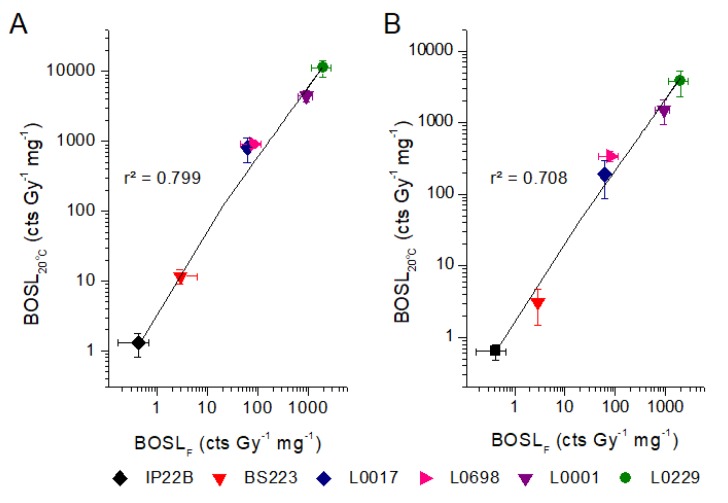
Comparison between BOSL_F_ sensitivity and sensitivity measured at room temperature (BOSL_20°C_). (**A**) BOSL_20°C_ sensitivity measured without pause between dose and blue stimulation. (**B**) BOSL_20°C_ sensitivity measured with 16 h pause between dose and blue stimulation. Each sample is represented by the average of three aliquots. Error bars correspond to the standard deviation.

The BOSL_20°C_ sensitivity measured at room temperature has positive correlation with the BOSL_F_ (preheat at 190 °C). The correlation is observed both for the sensitivity measured with no pause or with pause of 16 h before blue stimulation (Figure 4), which confirms that the OSL sensitivity obtained without use of thermal treatments to eliminate unstable components can be used for quartz fingerprinting. The BOSL_20°C_ sensitivity obtained for measurements after 16 h pause is two- to three-times lower than measured without pause (Table 5), confirming the significant contribution of unstable OSL components.

**Figure 5:**
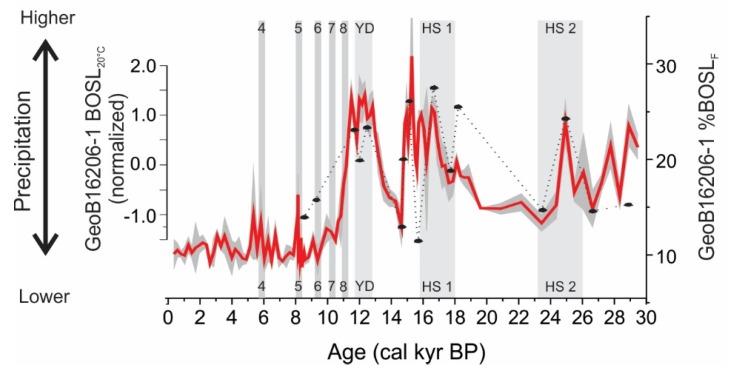
OSL sensitivity for GeoB16206-1 sediment core, with standard error (gray shading) [30] and normalized BOSL_20°C_ (dashed black line, this study). Bond events 4, 5, 6, 7, and 8, Younger Dryas (YD), and the Heinrich Stadials (HS) 1 and 2 represent periods of higher precipitation in Northeast Brazil [33,34].

Polymineral fine-grained aliquots of core GeoB16206-1 show BOSL_20°C_ sensitivity measured at room temperature have a significant correlation (r^2^ = 0.56) with the BOSL_F_ sensitivity measured with stimulation at 125 °C (Table 7). The BOSL_20°C_ sensitivity at room temperature also reproduces the pattern that links OSL sensitivity and precipitation changes (Figure 5).

## 4. Discussion

When quartz OSL is measured by using preheat (160–260 °C), blue stimulation at 125 °C, and light detection in the ultraviolet band, the initial 1 s of light emission is presumably dominated by the fast OSL component [26], which was intensely characterized for dating purposes [25,35]. The extensive research to develop dating protocols based on the fast OSL component of quartz has also supported applications in sediment provenance studies, e.g., [21,22,23,24,27,28]. However, thermal treatments to isolate the fast OSL component usually require mounting samples on discs or cups for measurements in OSL readers built for dosimetry purposes. The decrease of the preheat temperature from 400 to 50 °C allows us to increase the intensity of the OSL signal (initial 1 s) and still preserve the pattern of sensitivity variation observed in the studied quartz samples using protocols [23] designed to measure a signal dominated by the fast OSL component. Higher signal intensity acquired by using lower preheat temperature is advantageous to measure low-sensitivity samples because it permits the use of lower given doses and reduce measurement time. In the same way, the OSL sensitivity measured without preheating and with stimulation at room temperature (~20 °C) is positively correlated with the sensitivity of the OSL signal with higher contribution of the fast component. The OSL sensitivities at room temperature (BOSL_20°C_) recorded after a 16 h pause or without pause between dosing and stimulation have similar patterns of variation. Accordingly, the preheat to eliminate unstable OSL components and light stimulation at 125 °C to avoid any effect of the 110 °C TL on OSL signal measurement can be neglected in OSL sensitivity assessment for provenance analysis of sediments. The BOSL_20°C_ signal measured without preheating has higher contribution of medium and slow components, which are partially removed if heating treatments are applied before light stimulation [26]. Its correlation with the BOSL_F_ sensitivity agrees with the observation that low and medium OSL components are also sensitized in nature (bleaching–irradiation cycles), similarly to the fast OSL component [13,20]. The main advantages of the BOSL_20°C_ measured without preheating are the reduction of measurement time and the possibility to perform in situ measurements on sediment cores or sedimentary successions exposed in outcrops, using portable OSL readers with an attached X-ray source, e.g., [36].

The assessment of quartz OSL sensitivity in polymineral aliquots of sand requires bleaching other minerals presenting BOSL signals, but preserving the quartz OSL signal. Besides quartz, main mineral components of sands are feldspar and heavy minerals. Feldspar OSL signals can be bleached by using infrared stimulation, allowing for the measurement of quartz OSL signals in the presence of feldspar [37]. However, little is known about the effect of heavy minerals for measurement of quartz OSL sensitivity in polymineral aliquots. Magnetic heavy mineral concentrates have OSL sensitivities similar to low-sensitivity quartz, while nonmagnetic heavy minerals can show OSL sensitivities similar to medium-sensitivity quartz (Figure 1 and Table 5). Heavy minerals are minor components of sands, with higher concentrations (>10%) occurring only in sediments from specific geological settings, such as in areas around mafic parent rocks or environments where hydraulic conditions promoted the selective transport of lighter minerals like quartz and feldspar [38]. Thus, OSL signals arising from heavy minerals have minor influence on the record of quartz OSL sensitivity in bulk samples. However, the tracking of sediment layers enriched in heavy minerals (placers) is recommended to validate that the bulk OSL signal is dominated by quartz. This can be performed by using elemental composition characterized through portable X-ray fluorescence spectrometers [39].

Quartz OSL sensitivity (BOSL_F_) measured in polymineral fine-grained sediments (silt) can also be applied in provenance analysis, e.g., [23]. A recent example of a successful application of OSL sensitivity for provenance analysis comprised measurements in silt of a marine sediment core [30]. Marine sediment cores are one of the most commonly used archives for the reconstruction of past climatic and oceanographic changes, e.g., [40,41,42,43,44]. The OSL sensitivity represented by the percentage of the initial (1 s) light emission in relation to the total OSL emission (%BOSL_F_) was used to track changes in precipitation over the Parnaíba River watershed in Northeastern Brazil [28], where higher values of sensitivity resemble with moments of increased precipitation (i.e., Heinrich Stadials 2 and 1, and the Younger Dryas) [36]. Our data show that BOSL sensitivity measured at room temperature (BOSL_20°C_) in silt samples of core GeoB16206-1 can be used in the same way as the BOSL_F_ signal with stimulation at 125 °C (Figure 3 and Figure 5). This allows us to increase data resolution through in situ measurements and to couple the provenance analysis of different grain-size fractions (silt and sand), using a single mineral component (quartz). Provenance analysis based on a single and chemically resistant mineral type avoids spurious effects associated with hydraulic sorting during sediment transport or with compositional changes due to weathering or diagenesis.

Therefore, the quartz OSL sensitivity measured without preheating and using blue light stimulation at room temperature after a given dose is recommended for provenance analysis of quartz. Future research can evaluate the use of other ionizing radiation sources, such as portable X-ray sources, and test in situ OSL-sensitivity measurements, using a similar approach as employed, for example, in bulk sediment geochemistry, using X-ray fluorescence. In this case, it is also necessary to improve protocols to measure quartz OSL signals in polymineral material, especially in the presence of feldspar [37], but without the use of thermal treatments.

## Figures and Tables

**Table 1:** Summary of characteristics of quartz samples used in this study.

Code	Grain Size	* OSL Sensitivity	Source Material	Geological Setting
IP22B	180–250 µm	Low	Hydrothermal vein	Paraná Basin (Brazil)
BS223	180–250 µm	Low	Blue schist	Andes (Chile)
L0674	180–250 µm	Low	Alluvial sand	Atacama (Chile)
L0017	180–250 µm	Medium	Fluvial sand	Central Amazon
L0698	63–180 µm	Medium	Fluvial sand	Western Amazon
L0001	180–250 µm	High	Coastal sand	Santa Catarina coast
L0229	180–250 µm	High	Fluvial sand	Pantanal Wetland
L0231	180–250 µm	High	Fluvial sand	Pantanal Wetland
L0688	180–250 µm	High	Fluvial sand	Paraná River basin
** Cal. Qz	180–250 µm	Very high	Eolian sand	Jutland, Denmark

* OSL sensitivity appraised in a previous study [14]. ** “Cal. Qz” is quartz used for calibration of beta radiation sources of luminescence readers. It was artificially sensitized by heating at 700 °C for 1 h, 2 kGy gamma dose, and another heating at 450 °C for 1 h [30].

**Table 2:** Sequence of procedures used to measure OSL sensitivity of quartz and heavy mineral aliquots. BOSL_F_ represents the signal used for sensitivity calculation, which was measured under varied preheat conditions (50–400 °C) and using blue stimulation at 125 °C.

Step	Treatment	Observed
1	Illumination with blue LED at 125 °C for 100 s	
2	Give dose, 50 Gy	
3	(1) Preheat at 190 °C for 10 s, (2) 50, 100, 150, 200, 300, or 400 °C for 10 s	
4	Infrared LED stimulation at 60 °C for 300 s	
5	Blue LED stimulation at 125 °C for 100 s	BOSL_F_

**Table 3:** Sequence of procedures used to measure OSL sensitivity of pure quartz aliquots without preheating and with light stimulation at room temperature (20 °C). BOSL_20°C_ and BOSL_125°C_ represent the signals used for sensitivity calculation.

Step	Treatment	Observed
1	Illumination with blue LED at 20 °C for 500 s	
2	Give dose, 50 Gy	
3	Pause for 0 s or 16 h	
4	Blue LED stimulation at 20 °C for 100 s	BOSL_20°C_
5	Blue LED stimulation at 125 °C for 100 s	BOSL_125°C_

**Table 4:** Sequence of procedures to measure BOSL_20°C_ sensitivity in aliquots of fine-grained sediments from core GeoB16206-1.

Step	Treatment	Observed
1	Illumination with blue LED at 125 °C for 100 s	
2	Give dose, 10 Gy	
3	Blue LED stimulation at 20 °C for 100 s	BOSL_20°C_

**Table 5:** Average (*n* = 3 aliquots) OSL sensitivity (± standard deviation) of studied quartz samples. The OSL sensitivity of heavy minerals concentrates from coastal sands (samples L0387 and L0393) are also shown for comparison. The OSL sensitivities of magnetic (M) and nonmagnetic (NM) heavy minerals were calculated in the same way as the BOSL_F_. OSL signal was not detected for the magnetic heavy mineral fraction of sample L0387. A given dose of 50 Gy was used for all aliquots.

Sample	%BOSL_F_	BOSL_F_	BOSL_125°C_/0 s	BOSL_125°C_/16 h	BOSL_20°C_/0 s	BOSL_20°C_/16 h
		(cts Gy^−1^ mg^−1^)	(cts Gy^−1^ mg^−1^)	(cts Gy^−1^ mg^−1^)	(cts Gy^−1^ mg^−1^)	(cts Gy^−1^ mg^−1^)
IP22B	<1.00 ± 4.70	0.42 ± 0.25	<0.10 ± 0.17	<0.10 ± 0.15	1.31 ± 0.49	0.64 ± 0.16
BS223	8.43 ± 5.03	2.89 ± 3.34	1.47 ± 0.52	0.87 ± 0.32	11.74 ± 2.77	3.11 ± 1.65
L0674	12.33 ± 4.83	11.20 ± 7.36	-	-	-	-
L0017	19.36 ± 0.17	62.60 ± 4.98	55.68 ± 11.95	46.29 ± 8.60	814.58 ± 317.15	189.21 ± 101.99
L0698	19.40 ± 4.46	80.41 ± 35.04	52.61 ± 5.84	51.81 ± 3.52	917.67 ± 113.41	341.72 ± 58.18
L0001	50.28 ± 6.74	925.57 ± 295.55	94.91 ± 21.50	118.38 ± 25.81	4501.14 ± 807.25	1468.82 ± 566.53
L0229	44.78 ± 2.16	1963.84 ± 835.53	230.30 ± 43.89	254.61 ± 44.12	11,376.18 ± 3089.01	3794.16 ± 1489.61
L0231	36.31 ± 11.03	1311.64 ± 401.13	-	-	-	-
L0688	54.52 ± 6.48	3370.86 ± 205.27	-	-	-	-
CalQz	56.08 ± 2.94	8565.00 ± 497.76	-	-	-	-
L0387_NM_	25.75 ± 6.28	20.80 ± 6.00	-	-	-	-
L0393_M_	57.79 ± 49.30	1.19 ± 0.65	-	-	-	-
L0393_NM_	33.31 ± 25.88	94.77 ± 103.03	-	-	-	-

**Table 6:** Quartz BOSL_F_ sensitivity measured using different preheat temperatures (step 5 of the protocol in Table 2). Sensitivity values correspond to the average of three aliquots (± standard deviation). Sample type is described in Table 1.

Preheat	BOSL_F_ Sensitivity (cts Gy^−1^ mg^−1^)		
	IP22B	L0017	L0229
50 °C	1.60 ± 1.12	111.24 ± 71.03	2773.75 ± 1858.26
100 °C	1.15 ± 0.90	75.58 ± 10.31	4723.34 ± 4264.40
150 °C	0.41 ± 0.45	62.11 ± 9.11	2252.60 ± 512.44
200 °C	0.20 ± 0.18	36.30 ± 7.98	2227.47 ± 694.52
300 °C	0.19 ± 0.09	34.09 ± 9.75	981.85 ± 161.90
400 °C	<0.10 ± 0.27	0.84 ± 028	0.66 ± 064

**Table 7:** Comparison between BOSL_F_ sensitivity [30] and sensitivity measured at room temperature (BOSL_20°C_) for silt aliquots from GeoB16206-1 sediment core. Sensitivity values correspond to the average of three aliquots (± standard deviation). Data normalization included subtraction of mean and division by the standard deviation of the data set (%BOSL_20°C_ or %BOSL_F_).

Sample Depth (cm)	%BOSL_20°C_	BOSL_20°C_ (Normalized)	%BOSL_F_	BOSL_F_ (Normalized)
96	14 ± 2	−1.01	9 ± 1	−1.78
150	22 ± 9	−0.66	12 ± 1	−1.34
260	53 ± 5	0.75	27 ± 2	0.99
280	39 ± 5	0.13	22 ± 1	0.31
310	54 ± 7	0.79	27 ± 1	1.04
400	10 ± 2	−1.20	13 ± 2	−1.11
430	40 ± 5	0.16	25 ± 3	0.72
460	65 ± 2	1.31	31 ± 8	1.68
490	4 ± 1	−1.48	19 ± 2	−0.17
550	71 ± 1	1.58	26 ± 1	0.83
610	35 ± 5	−0.08	18 ± 2	−0.40
630	63 ± 3	1.20	20 ± 1	−0.05
700	18 ± 3	−0.85	13 ± 1	−1.05
720	58 ± 9	0.97	24 ± 2	0.60
750	17 ± 4	−0.88	15 ± 2	−0.82
790	20 ± 1	−0.75	24 ± 2	0.54

## References

[1] Meunier P., Hovius N., Haines J. (2007). Regional patterns of earthquake-triggered landslides and their relation to ground motion. Geophys. Res. Lett..

[2] Chen H., Dadson S., Chi Y.-G. (2006). Recent rainfall-induced landslides and debris flow in northern Taiwan. Geomorphology.

[3] Stuut J.-B., Zabel M., Ratmeyer V., Helmke P., Schefuß E. (2005). Provenance of present-day eolian dust collected off NW Africa. J. Geoph. Res..

[4] Goodbred S.L. (2003). Response of the Ganges dispersal system to climate change: A source-to-sink view since the last interstade. Sed. Geol..

[5] Yang S.Y., Jung H.S., Lim D.I., Li C.X. (2003). A review on the provenance discrimination of sediments in the Yellow Sea. Earth Sci. Rev..

[6] Martin S.J., Funch R.R., Hanson P.R., Yoo E.-H. (2018). A vast 4000-year-old spatial pattern of termite mounds. Curr. Biol..

[7] Allan James L. (2013). Legacy sediment: Definitions and processes of episodically produced anthropogenic sediment. Anthropocene.

[8] Bond G., Broecker W., Johnsen S., McManus J., Labeyrie L., Jouzel J., Bonani G. (1993). Correlations between climate records from North Atlantic sediments and Greeland ice. Nature.

[9] Concheri G., Bertoldi D., Polone E., Otto S., Larcher R., Squartini A. (2011). Chemical elemental distribution and soil DNA fingerprints provide the critical evidence in murder case investigation. PLoS ONE.

[10] Dickinson W.R., Zuffa G.G. (1985). Interpreting provenance relations from detrital modes of sandstones. Provenance of Arenites.

[11] Morton A., Morton A.C., Todd S.P., Haughton P.D.W. (1991). Geochemical studies of detrital heavy minerals and their application to provenance research. Developments in Sedimentary Provenance Studies.

[12] Gray H.J., Jain M., Sawakuchi A.O., Mahan S.A., Tucker G.E. (2019). Luminescence as a sediment tracer and provenance tool. Rev. Geophys..

[13] Sawakuchi A.O., Blair M.W., De Witt R., Faleiros F.M., Hyppolito T., Guedes C.C.F. (2011). Thermal history versus sedimentary history: OSL sensitivity of quartz grains extracted from rocks and sediments. Quat. Geochronol..

[14] Mineli T.D., Sawakuchi A.O., Guralnik B., Lambert R., Jain M., Pupim F.N., del Río I., Guedes C.C.F., Nogueira L. (2020). Variation of luminescence sensitivity, characteristic dose and trap parameters of quartz. Radiat. Meas..

[15] Bøtter-Jensen L., Thomsen K.J., Jain M. (2010). Review of optically stimulated luminescence (OSL) instrumental developments for retrospective dosimetry. Radiat. Meas..

[16] Richter D., Richter A., Dornich K. (2013). Lexsyg—A new system for luminescence research. Geochronometria.

[17] Kook M., Lapp T., Murray A.S., Thomsen K.J., Jain M. (2015). A luminescence imaging system for the routine measurement of single-grain OSL dose distributions. Radiat. Meas..

[18] Guralnik B., Ankjærgaard C., Jain M., Murray A.S., Müller A., Wällea M., Lowick S.E., Preusser F., Rhodes E.J., Wu T.-S. (2015). OSL-thermochronometry using bedrock quartz: A note of caution. Quat. Geochronol..

[19] Bøtter-Jensen L., Agersnap Larsen N., Mejdahl V., Poolton N.R.J., Morris M.F., McKeever S.W.S. (1995). Luminescence sensitivity changes in quartz as a result of annealing. Rad. Meas..

[20] Moska P., Murray A.S. (2006). Stability of the quartz fast-component in insensitive samples. Radiat. Meas..

[21] Tsukamoto S., Nagashima K., Murray A.S., Tada R. (2011). Variations in OSL components from quartz from Japan sea sediments and the possibility of reconstructing provenance. Quat. Int..

[22] Pietsch T.J., Olley J.M., Nanson G.C. (2008). Fluvial transport as a natural luminescence sensitiser of quartz. Quat. Geochronol..

[23] Sawakuchi A.O., Jain M., Mineli T.D., Nogueira L., Bertassoli D.J., Häggi C., Sawakuchi H.O., Pupim F.N., Grohmann C.H., Chiessi C.M. (2018). Luminescence of quartz and feldspar fingerprints provenance and correlates with the source area denudation in the Amazon River basin. Earth Planet. Sci. Lett..

[24] Lü T., Sun J., Li S.-H., Gong Z., Xu L. (2014). Vertical variations of luminescence sensitivity of quartz grains from loess/paleosol of Luochuan section in the central Chinese Loess Plateau since the last interglacial. Quat. Geochronol..

[25] Murray A.S., Wintle A.G. (2003). The single aliquot regenerative dose protocol: Potential for improvements in reliability. Radiat. Meas..

[26] Jain M., Murray A.S., Bøtter-Jensen L. (2003). Characterization of blue-light stimulated luminescence components in different quartz samples: Implications for dose measurement. Radiat. Meas..

[27] Zular A., Sawakuchi A.O., Guedes C.C., Giannini P.C.F. (2015). Attaining provenance proxies from OSL and TL sensitivities: Coupling with grain size and heavy minerals data from southern Brazilian coastal sediments. Radiat. Meas..

[28] Mendes V.R., Sawakuchi A.O., Chiessi C.M., Giannini P.C.F., Rehfeld K., Mulitza S. (2019). Thermoluminescence and optically stimulated luminescence measured in marine sediments indicate precipitation changes over northeastern Brazil. Paleoceanography.

[29] Mulitza S., Chiessi C.M., Cruz A.P.S., Frederichs T.W., Gomes J.G., Gurgel M.H.C., Haberkern J., Huang E., Jovane L., Kuhnert H. (2012). Response of Amazon Sedimentation to Deforestation, Land Use and Climate Variability—Cruise No. MSM20/3—Recife, Brazil–Bridgetown, Barbados, 19 February–11 March 2012. MARIA S. MERIAN-Berichte, MSM20/3.

[30] Hansen V., Murray A.S., Buylaert J.P., Yeo E.Y., Thomsen K. (2015). A new irradiated quartz for beta source calibration. Radiat. Meas..

[31] Duller G.A.T. (2015). The Analyst software package for luminescence data: Overview and recent improvements. Anc. TL.

[32] Timar A., Vandenberghe D., Panaiotu E.C., Panaiotu C.G., Necula C., Cosma C., van den Haute P. (2010). Optical dating of Romanian loess using fine-grained quartz. Quat. Geochronol..

[33] Stríkis N.M., Cruz F.W., Cheng H., Karmann I., Edwards R.L., Vuille M., Wang X., de Paula M.S., Novello V.F., Auler A.S. (2011). Abrupt variations in South American monsoon rainfall during the Holocene based on a speleothem record from central-eastern Brazil. Geology.

[34] Mulitza S., Chiessi C.M., Schefuß E., Lippold J., Wichmann D., Antz B., Mackensen A., Paul A., Prange M., Rehfeld K. (2017). Synchronous and proportional deglacial changes in Atlantic meridional overturning and northeast Brazilian precipitation. Paleoceanography.

[35] Wintle A.G., Murray A.S. (2006). A review of quartz optically stimulated luminescence characteristics and their relevance in single-aliquot regeneration dating protocols. Radiat. Meas..

[36] Blair M.W., Yukihara E.G., McKeever S.W.S. (2006). A system to irradiate and measure luminescence at low temperatures. Radiat. Prot. Dosim..

[37] Wallinga J., Murray A.S., Bøtter-Jensen L. (2002). Measurement of the dose in quartz in the presence of feldspar contamination. Radiat. Prot. Dosim..

[38] Garzanti E., Andò S. (2007). Heavy mineral concentration in modern sands: Implications for provenance interpretation. Develop. Sedim..

[39] Ryan J.G., Shervais J.W., Li Y., Reagan M.K., Li H.Y., Heaton D., Godard M., Kirchenbaur M., Whattam S.A., Pearce J.A. (2017). Application of a handheld X-ray fluorescence spectrometer for real-time, high density quantitative analysis of drilled igneous rocks and sediments during IODP Expedition 352. Chem. Geol..

[40] Molfino B., McIntyre A. (1990). Precessional forcing of nutricline dynamics in the equatorial Atlantic. Science.

[41] Duplessy J.C., Labeyrie L., Juillet-Leclerc A., Maitre F., Duprat J., Sarnthein M. (1992). Surface salinity reconstruction of the North Atlantic Ocean during the last glacial maximum. Oceanol. Acta.

[42] Nürnberg D., Bijma J., Hemleben C. (1996). Assessing the reliability of magnesium in foraminiferal calcite as a proxy for water mass temperatures. Geochim. Cosmochim. Acta.

[43] Mulitza S., Dürkoop A., Hale W., Wefer G., Stefan Niebler H. (1997). Planktonic foraminifera as recorders of past surface-water stratification. Geology.

[44] Govin A., Holzwarth U., Heslop D., Ford Keeling L., Zabel M., Mulitza S., Collins J.A., Chiessi C.M. (2012). Distribution of major elements in Atlantic surface sediments (36 N–49 S): Imprint of terrigenous input and continental weathering. Geochem. Geophy. Geosy..

